# Gene Regulation and Mitochondrial Activity During White and Brown Adipogenesis Are Modulated by KDM5 Histone Demethylase

**DOI:** 10.1210/jendso/bvae029

**Published:** 2024-02-13

**Authors:** Laurent Vergnes, Carrie B Wiese, Temeka Zore, Carrie Riestenberg, Rozeta Avetisyan, Karen Reue

**Affiliations:** Department of Human Genetics, David Geffen School of Medicine at UCLA, Los Angeles, CA 90095, USA; Department of Human Genetics, David Geffen School of Medicine at UCLA, Los Angeles, CA 90095, USA; Department of Human Genetics, David Geffen School of Medicine at UCLA, Los Angeles, CA 90095, USA; Department of Human Genetics, David Geffen School of Medicine at UCLA, Los Angeles, CA 90095, USA; Department of Human Genetics, David Geffen School of Medicine at UCLA, Los Angeles, CA 90095, USA; Department of Human Genetics, David Geffen School of Medicine at UCLA, Los Angeles, CA 90095, USA; Molecular Biology Institute, University of California, Los Angeles, CA 90095, USA

**Keywords:** adipocyte differentiation, mitochondrial respiration, gene expression, KDM5C

## Abstract

Body fat accumulation differs between males and females and is influenced by both gonadal sex (ovaries vs testes) and chromosomal sex (XX vs XY). We previously showed that an X chromosome gene, *Kdm5c*, is expressed at higher levels in females compared to males and correlates with adiposity in mice and humans. *Kdm5c* encodes a KDM5 histone demethylase that regulates gene expression by modulating histone methylation at gene promoters and enhancers. Here, we use chemical inhibition and genetic knockdown to identify a role for KDM5 activity during early stages of white and brown preadipocyte differentiation, with specific effects on white adipocyte clonal expansion, and white and brown adipocyte gene expression and mitochondrial activity. In white adipogenesis, KDM5 activity modulates H3K4 histone methylation at the *Dlk1* gene promoter to repress gene expression and promote progression from preadipocytes to mature adipocytes. In brown adipogenesis, KDM5 activity modulates H3K4 methylation and gene expression of *Ucp1*, which is required for thermogenesis. Unbiased transcriptome analysis revealed that KDM5 activity regulates genes associated with cell cycle regulation and mitochondrial function, and this was confirmed by functional analyses of cell proliferation and cellular bioenergetics. Using genetic knockdown, we demonstrate that KDM5C is the likely KDM5 family member that is responsible for regulation of white and brown preadipocyte programming. Given that KDM5C levels are higher in females compared to males, our findings suggest that sex differences in white and brown preadipocyte gene regulation may contribute to sex differences in adipose tissue function.

Obesity affects approximately 40% of adults in the United States and rates continue to rise (www.cdc.gov). Obesity is a risk factor for numerous disease states, including cardiovascular disease, type 2 diabetes, nonalcoholic fatty liver, and some types of cancers. Several important differences exist between men and women in the storage and utilization of lipids in adipose tissue [1-4]. In general, men are more likely than women to store fat in visceral depots, and men are better able to mobilize stored fat than women. Women, on the other hand, have higher proportional body fat stores than men and are more likely to store fat in the gluteo-femoral region.

Mechanisms that influence sex differences in adiposity include both gonadal hormones (from ovaries or testes) and chromosomal sex (XX or XY chromosomes) [5]. Ovarian hormones have a major impact on fat storage, as established by studies in pre- and postmenopausal women, and in rodents before and after removal of gonads. In general, reduced ovarian hormone levels in females are associated with increased visceral adiposity, insulin resistance, dyslipidemia, and risk for cardiovascular disease [6, 7]. Additionally, male and female mice lacking the estrogen receptor have increased white adipose tissue mass and increased insulin resistance [8]. In contrast, estrogen replacement in postmenopausal women reduces central adipose tissue mass [9]. However, not all sex differences in adiposity can be attributed to gonadal hormones. For example, sex differences exist in body composition of human and mouse embryos prior to the action of gonadal hormones, indicating that sex chromosome complement likely plays a role [10, 11]. Furthermore, studies performed in a mouse model that allows the distinction between gonadal and chromosomal sex contributions revealed that XX compared to XY chromosome complement promotes enhanced adipose tissue accumulation independent of gonadal hormones [12, 13].

We previously identified the X chromosome gene *Kdm5c* as a key determinant of adiposity in the mouse [13]. Due to its escape from X chromosome inactivation, *Kdm5c* is expressed at higher levels in the adipose tissues of XX compared to XY mice [12, 13] and *KDM5C* is expressed at higher levels in women compared to men [14]. In our previous study, we demonstrated that *Kdm5c* dosage correlates positively with fat mass and expansion in response to a high-fat diet in mice, and that human *KDM5C* expression levels correlate with body mass index [13]. The KDM5C protein is an H3K4 (histone 3, lysine 4) demethylase enzyme that modulates histone methylation status at gene transcription start sites and enhancers to regulate gene expression [15, 16].

KDM5C belongs to a family of 4 KDM5 histone demethylase enzymes, which also includes KDM5A, KDM5B, and KDM5D [17]. In both mouse and human, the genes encoding KDM5A and KDM5B are located on autosomes and are expressed at similar levels in males and females. By contrast, KDM5C and KDM5D are encoded on the X and Y chromosomes, respectively, and have differential expression levels between male and female humans and mice [18, 19]. All KDM5 family members are presumed to catalyze the conversion of H3K4 trimethyl (me3) to H3K4 di- and mono-methyl forms (me2 and me). Previous studies of KDM5 enzymes in adipogenesis demonstrated roles for KDM5A and KDM5C in adipocyte differentiation using genetic knockdown in white preadipocytes or genetic haploinsufficiency in the mouse [13, 20]. However, these studies did not determine the temporal dynamics of KDM5 enzyme action during white adipogenesis, and no studies have assessed a potential role for KDM5 histone demethylases in brown adipogenesis.

Here, we determine that KDM5 histone-modifying activity is critical in a temporally defined manner to establish white and brown preadipocyte gene expression programs that permit clonal expansion, induction of adipogenic transcription factors and lipogenic genes, and mitochondrial respiration adaptation during adipocyte differentiation. KDM5 effects on gene expression during adipogenesis correlate with regulation of H3K4me3 histone marks at genes critical in the progression of white and brown adipocyte differentiation. Through analysis of protein levels and genetic knockdown, we identify KDM5C as the likely KDM5 family member responsible for epigenetic regulation of key genes that allow progression of white preadipocytes to mature adipocytes, and for induction of thermogenic genes in brown adipocytes. These findings illuminate mechanisms underlying our previous observation that *Kdm5c* gene dosage modulates adiposity in vivo [13].

## Materials and Methods

### Cell Culture

Mouse 3T3-L1 cells (passages 6-12) and medium were obtained from ZenBio (Durham, NC) and were cultured and induced to differentiate according to the manufacturer's protocol with minor changes. Briefly, 3T3-L1 cells were plated in preadipocyte medium (DMEM, containing 10% calf serum) and cultured to confluence (defined as day −2). Two days after confluence (defined as day 0), cells were induced to differentiate using a medium containing dexamethasone, insulin, isobutylmethylxanthine, and PPARγ agonist. Cells were maintained in differentiation medium for 2 days and then switched to an adipocyte maintenance medium containing insulin and dexamethasone, which was changed every other day. Immortalized brown adipocyte cells were a gift from Bruce Spiegelman [21] and were differentiated as previously described [22]. Cells were treated at specified time points with 10 µM KDM5-C70, a chemical inhibitor of KDM5 histone demethylase activity (Xcess Biosciences, Inc., Chicago, IL) or DMSO vehicle alone.

### Oil Red O Staining and Quantification

Neutral lipid staining and quantification were performed with Oil Red O (ORO, 0.2% w/v) as described previously [13].

### Cell Proliferation

Cells were plated in 12-well plates and treated with C70 at confluence (day −2); they were stimulated to differentiate at day 0 as described above. At indicated time points (days 0 through 4), cells were collected by trypsinization and counted either with a hemocytometer or by fluorescence with Hoechst 33342 dye in an Operetta (Perkin Elmer, Shelton, CT). For genomic DNA quantification, trypsinized cells were resuspended in TNE buffer (10 mM Tris, pH 7.4, 100 mM NaCl, 1 mM EDTA), and incubated with 1 µg/mL Hoechst 33342. A standard curve was obtained using a pool of DNA diluted in TNE. Fluorescence was measured with a VersaFluor fluorometer (Bio-Rad, Hercules, CA).

### RNA Extraction, cDNA Synthesis, and Real-Time Quantitative Polymerase Chain Reaction

RNA was extracted with Trizol (Invitrogen, Waltham, MA). cDNA synthesis was performed using iScript Reverse Transcription Supermix (Bio-Rad) and quantitative polymerase chain reaction (qPCR) was performed on a CFX Connect (Bio-Rad) using iTaq Universal SYBR Green Supermix (Bio-Rad). Primers for qPCR are listed in Supplementary Table S1 [23].

### Immunoblots

Cellular protein (30 µg) was electrophoresed on SDS-polyacrylamide gels and transferred to a nitrocellulose membrane. After blocking with 5% milk in TBST (0.1% Tween 20 in TBS), the membrane was incubated with primary antibody (1:1000) overnight, followed by secondary antibody (1:10 000) for 1 hour, with TBST washes in between. Antibodies used for Western blots: KDM5A (also known as JARID1A; RRID: AB_2234038), KDM5B (also known as JARID1B; RRID: AB_1264323), KDM5C (also known as JARID1C; RRID: AB_2615064), alpha Tubulin (RRID: AB_10722892), and anti-rabbit IgG (RRID: AB_2533967). Immunoreactive bands were developed with ECL Prime (RPN2232, Millipore, Burlington, MA) and visualized with a Bio-Rad Gel-Doc imager.

### Plasmids and siRNA Transfection

Expression plasmids for *Ccna2* and *Ccne1* were purchased from Origene. The gene coding regions were cloned into a pCMV6 vector with a C-terminal Myc tag. Cells were transfected with BioT (Bioland Scientific, Paramount, CA) following the manufacturer's protocol. The siRNA knockdown was described previously [13]. Briefly, 50 nM of ON-TARGETplus siRNA (Horizon Discovery, St. Louis, MO) was reverse-transfected with Lipofectamine RNAi-MAX (Thermo Fisher Scientific, Waltham, MA).

### RNA-seq Analysis

Library construction, sequencing and alignment were performed at the Technology Center for Genomics & Bioinformatics at UCLA as described [13]. Briefly, read data were aligned by STAR (version 2.7.2a) to Ensembl reference genome GRCm38.97. Differentially expressed genes (DEG) were identified by EdgeR (v3.28.1) using trimmed mean of M values normalization and significance as adjusted *P* value < .05 (Robinson 2010, McCarthy 2012). The DEG with > 1.25-fold change and adjusted *P* value < .05 for at least one time point were soft clustered with the Mfuzz package (v2.46.0) [24]. A total of 8 clusters were generated with a 2.0 fuzzification parameter and membership >0.5.

### Chromatin Immunoprecipitation

Cells were fixed for 10 minutes with 1% formaldehyde and quenched with 12.5 mM glycine at room temperature. Nuclei were isolated in a hypotonic buffer (20 mM Tris-HCL, 10 mM NaCl, 3 mM MgCl_2_) with Dounce homogenization. The chromatin was sheared by sonication (5 cycles of 15 seconds) in 1% SDS buffer with average fragment size of 200 to 600 bp. The soluble chromatin was incubated overnight with primary antibodies at 4 °C. The immunoprecipitation was performed with protein A/G plus agarose beads (sc-2003, Santa Cruz Biotechnology, Dallas, TX) incubated for 1 to 2 hour at 4 °C and sequentially washed with low-salt buffer (0.1% SDS, 1% Triton X-100, 2 mM EDTA, 20 mM Tris-HCl, pH 8.1, 150 mM NaCl), high-salt buffer (0.1% SDS, 1% Triton X-100, 2 mM EDTA, 20 mM Tris-HCl, pH 8.1, 500 mM NaCl), LiCl buffer (1% NP40, 1% deoxycholate, 1 mM EDTA, 10 mM Tris-HCl, pH 8.1, 250 mM LiCl), and 1× TE buffer. The immune complexes were eluted with 1% SDS/0.1 M NaHCO_3_ and the cross-linking was reversed with 0.2 M NaCl treatment at 65 °C for > 4 hour. After RNase and proteinase K treatment, the DNA was recovered with PCR cleaning kit (Bioland Scientific). The qPCR was performed on a CFX Connect (Bio-Rad) using iTaq Universal SYBR Green Supermix (Bio-Rad), by calculating the fold-enrichment to the IgG isotype control after correcting for PCR efficiency for each primer set. Antibodies used for chromatin immunoprecipitation: rabbit anti-Histone H3 (RRID: AB_302613), rabbit anti-H3K4 monomethyl (RRID: AB_306847), rabbit anti-H3K4 trimethyl (RRID: AB_306649), and rabbit IgG isotype control (RRID: AB_2687657).

### Cellular Respiration

Cellular respiration was measured using a Seahorse XF24 or XF96 (Agilent Technologies, Santa Clara, CA), as described previously [22]. For 3T3-L1 cells, oxygen consumption rate (OCR) was measured before and after the sequential injection of 0.75 µM oligomycin, 0.5 µM FCCP and 0.75 μM rotenone/myxothiazol. For brown adipocytes, cells were activated with 10 nM CL316,243 1 hour prior to the assay, and OCR was measured before and after the sequential injection of 2 µM oligomycin, 2 µM FCCP and 2 μM rotenone/myxothiazol. Mitochondrial respiration was determined as the OCR difference between basal and rotenone/myxothiazol injections. ATP-linked respiration was determined as the oligomycin response. Proton leak was determined as the OCR difference between oligomycin and rotenone/myxothiazol injections. Following Seahorse assays, plates were fixed in formalin and incubated with 1.25 µg/mL Hoechst 33342 in PBS for 15 minutes to stain nuclei. After PBS washes, cell counts were determined using an Operetta high-content imaging system (Perkin Elmer) at 20× magnification.

### Statistical Analyses

Statistical analyses were performed in Prism (GraphPad Software, Boston, MA) as indicated in text and figure legends for each experiment. Analyses include repeated-measures ANOVA, 2-factor ANOVA, and unpaired 2-tailed Student *t* test for biochemical assays, and EdgeR and MFuzz packages for analysis of RNA-seq differential gene expression and clustering. A value of *P* < .05 was considered significant for ANOVA and *t* tests; criteria for significance in RNA-seq data is provided in the text.

## Results

### KDM5 Protein Levels Are Dynamic During Adipogenesis

**Figure 1.**
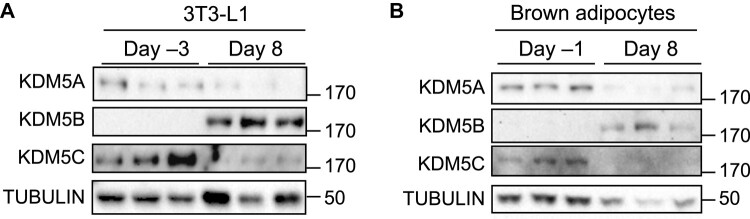
KDM5 protein levels in differentiating 3T3-L1 white adipocytes and brown adipocytes. (A) KDM5 protein detection by Western blot was performed in 3T3-L1 cells at the preadipocyte (day −3) and mature adipocyte stage (day 8). (B) KDM5 protein levels in brown preadipocytes (day −1) and mature adipocytes (day 8).

Previous studies have implicated KDM5 family members in adipogenesis and adipose tissue mass [13, 20], but the temporal regulation of KDM5 protein levels during adipocyte differentiation has not been established. We assessed the protein levels of KDM5 family members KDM5A, KDM5B, and KDM5C during white (3T3-L1) [25] and brown preadipocyte differentiation [21]. KDM5A and KDM5C proteins were present in white and brown preadipocytes (prior to confluence) but were diminished to very low levels in mature adipocytes (day 8 after differentiation induction) (Fig. 1A and B). Conversely, KDM5B protein was detectable only in mature white and brown adipocytes (Fig. 1A and B). Specific antibody for mouse KDM5D is not available, but *Kdm5d* mRNA was not detected in 3T3-L1 cells and was not investigated further. These findings indicate that KDM5A and KDM5C proteins are primarily present in preadipocytes, while KDM5B is detectable only in mature adipocytes.

### KDM5 Acts Exclusively During the Preadipocyte Stage to Influence White and Brown Adipogenesis

**Figure 2.**
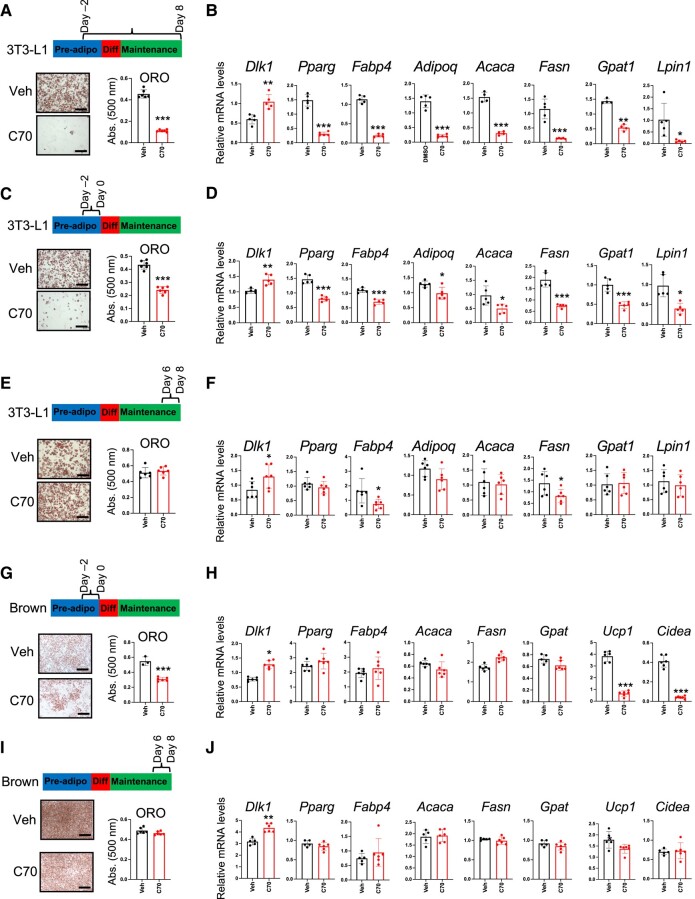
KDM5 influences adipocyte differentiation in a temporally restricted manner. 3T3-L1 and brown adipocyte differentiation was assessed by oil red O (ORO) staining of lipids and qPCR analysis of adipogenic and lipogenic gene expression. 3T3-L1 cells or brown adipocytes were treated with KDM5 inhibitor (C70) during the time intervals depicted on timelines. (A, B) C70 treatment of 3T3-L1 cells throughout differentiation, days −2 through 8. (C, D) C70 treatment of 3T3-L1 cells on days −2 to 0 only (prior to addition of differentiation medium). (E, F) C70 treatment of 3T3-L1 cells on days 6 through 8, after differentiation to mature adipocytes. (G, H) C70 treatment of brown adipocytes on days −2 to 0, prior to addition of differentiation medium. (I, J) C70 treatment of brown adipocytes on days 6 through 8, after differentiation to mature adipocytes. Comparison of vehicle- and C70-treated cells by *t* test; * *P* < .05; ** *P* < .01; *** *P* < .001.

We characterized the requirement for KDM5 activity at specific stages of white and brown adipogenesis by treating 3T3-L1 white preadipocytes and brown preadipocyte at defined intervals during differentiation with an inhibitor of KDM5 histone demethylase activity, KDM5-C70 (henceforth referred to as C70). In 3T3-L1 cells, C70 treatment beginning in preadipocytes and continuing throughout differentiation (day −2 through day 8) led to severely impaired mature adipocyte formation (Fig. 2A). This was apparent by a nearly complete lack of lipid accumulation (assessed by Oil Red O staining) and a transcriptional profile characteristic of preadipocytes, with elevated expression of the preadipocyte marker *Dlk1* (also known as *Pref1*), and low levels of adipogenic (*Pparg*, *Fabp4*, *Adipoq*) and lipogenic (*Acaca*, *Fasn, Gpat1, and Lpin1)* gene expression (Fig. 2B).

We next tested whether KDM5 inhibition in 3T3-L1 cells exclusively during the preadipocyte stage or exclusively in mature adipocytes would influence lipid accumulation and gene expression. 3T3-L1 preadipocytes treated with a single dose of C70 during a 2-day window prior to addition of differentiation medium (days −2 through 0) exhibited the same impairment of lipid accumulation and adipogenic/lipogenic gene expression as when treated for the duration of differentiation (Fig. 2C and D). By contrast, KDM5 inhibition in mature white adipocytes (days 6 through 8) led to normal lipid accumulation and only minor reductions in lipogenic gene expression, although *Dlk1* expression was elevated (Fig. 2E and F).

Similar temporal effects of KDM5 inhibition were observed in brown adipocytes, with treatment of preadipocytes (days −2 through 0) leading to reduced lipid accumulation and increased *Dlk1* expression, but unlike white preadipocytes, no effects on expression of *Pparg* or lipogenic genes (Fig 2G and 2H). Notably, KDM5 inhibition during the brown preadipocyte period prevented induction of the key thermogenic genes *Ucp1* and *Cidea*, which are a hallmark of thermogenic brown adipocytes (Fig. 2H). KDM5 inhibition in mature brown adipocytes (days 6 through 8) had no effect on lipid accumulation or expression of lipogenic or thermogenic genes (Fig. 2I and 2J). These results indicate that KDM5 activity is critical during the very early stage of white and brown preadipocyte commitment to form mature adipocytes, but does not substantially influence lipid accumulation or gene expression in mature adipocytes.

### KDM5 Modulates H3K4 Methylation at the *Dlk1* Promoter and *Ucp1* Enhancer

**Figure 3.**
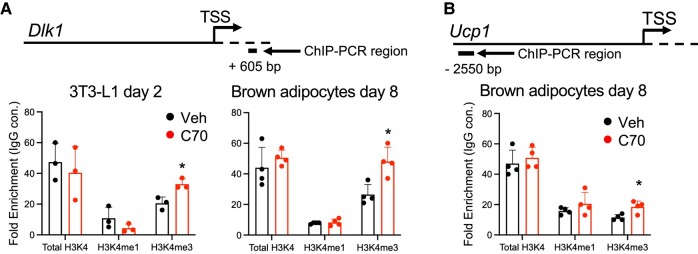
KDM5 modulates H3K4 methylation at the *Dlk1* promoter and *Ucp1* enhancer. H3K4 methylation status was determined by ChIP-qPCR at promoters/enhancers indicated with antibodies against total histone H3, lysine 4 trimethylated H3 (H3K4me3) and lysine 4 monomethylated H3 (H3K4me1). A control IgG antibody was used in tandem to assess nonspecific antibody binding and data are expressed as the fold-enrichment of H3 antibodies relative to the IgG control. (A) ChIP-qPCR of total H3 and H3K4 methylation status near the *Dlk1* transcription start site (+605 bp) in 3T3-L1 and brown adipocytes treated with KDM5 inhibitor C70 or vehicle (Veh) at day −2 and harvested for ChIP-qPCR at day 2 (3T3-L1) or treated with C70 at day −1 and harvested for ChIP-qPCR at day 8 (brown adipocytes). (B) ChIP-qPCR to assess total H3 and H3K4 methylation at *Ucp1* enhancer (−2550 bp) in brown adipocytes treated with C70 or Veh at day −1 and harvested for ChIP-qPCR at day 8. Comparison of Veh and C70-treated cells by *t* test; * *P* < .05.

Since KDM5 inhibition in both white 3T3-L1 and brown preadipocytes prevented the downregulation of *Dlk1* expression that normally occurs during differentiation, we hypothesized that the *Dlk1* gene is a direct target of KDM5 histone demethylase activity. The H3K4me3 histone mark at gene promoters generally enhances transcription, whereas it may repress transcription at enhancer regions [26]. We tested whether the enhanced *Dlk1* expression in preadipocytes treated with C70 reflects impaired H3K4me3 demethylation at the *Dlk1* promoter. We performed chromatin immunoprecipitation (ChIP)-PCR using primers near the *Dlk1* transcription start site (TSS). C70 treatment increased H3K4me3 marks near the *Dlk1* TSS in both 3T3-L1 and brown preadipocytes (Fig. 3A). These results suggest that the *Dlk1* promoter is a target of KDM5 demethylase activity, consistent with effects on gene expression during white and brown preadipocyte differentiation (see Fig. 1).

Based on the dramatic reduction in *Ucp1* expression in brown preadipocytes treated with C70 (Fig. 2H), we hypothesized that *Ucp1* may be a direct target of KDM5 activity. *Ucp1* gene expression is regulated by enhancer sequences located 2.5 Kb upstream of the TSS [27, 28]. C70 increased H3K4me3 marks at the *Ucp1* enhancer (Fig. 3B). Given that H3K4me3 marks at enhancers may repress transcription [26], this is consistent with the observed reduction in *Ucp1* expression in brown preadipocytes treated with KDM5 inhibitor (see Fig. 1).

### KDM5 Inhibition in 3T3-L1 White Preadipocytes Reduces Expression of Genes Associated With Cell Cycle and Mitochondrial Function

**Figure 4.**
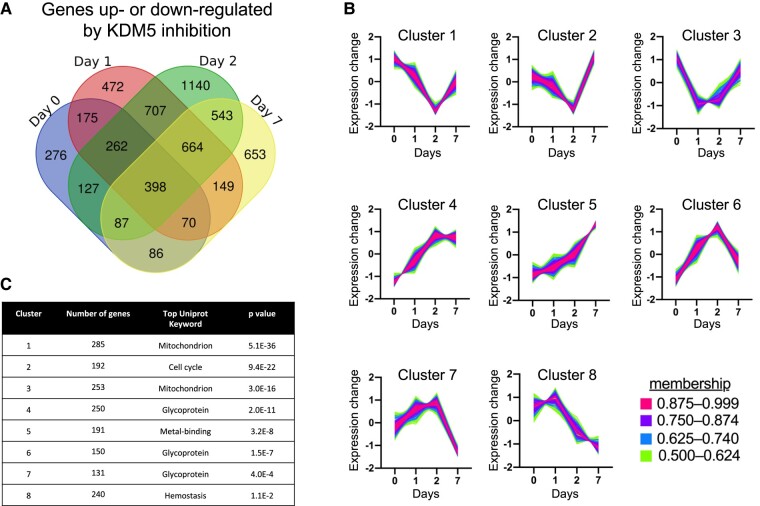
Temporal gene expression profiling after KDM5 inhibition in 3T3-L1 cells. 3T3-L1 preadipocytes were treated with vehicle or C70 at days −2 to 0, and harvested at days 0, 1, 2, and 7 of adipocyte differentiation for RNA-seq. Differentially expressed genes (DEG) were identified by EdgeR at adjusted *P* < .05. For clustering analysis by mFuzz, we included genes with > 1.25-fold change with C70 compared to vehicle and adjusted *P* < .05 for at least one time point. (A) Venn diagram illustrating the number of genes with altered expression in response to C70 treatment at each time point. Overlapping regions denote genes that exhibit altered expression at multiple time points. (B) Cluster analysis was performed using mFuzz to identify temporal gene expression patterns resulting from KDM5 inhibition. (C) Functional classification of genes contained in each mFuzz cluster was performed using the DAVID enrichment tool. The top Uniprot Keyword category for each cluster is shown with the corresponding adjusted *P* value (Benjamini correction).

To characterize the effect of KDM5 activity on preadipocyte gene expression in a global manner, we performed total RNA-sequencing of 3T3-L1 cells treated with C70 as preadipocytes (days −2 through 0) and collected at 4 time points throughout differentiation (days 0, 1, 2, and 7). We identified 5809 genes with altered expression in response to C70 (fold-change > 1.25 for at least one time point, adjusted *P* < .05) (Fig. 4A). Of these genes, 2543 genes (44%) were differentially expressed at more than one time point, and 398 genes were differentially expressed at all 4 time points after KDM5 inhibition. In addition, C70 treatment altered the expression of 2031 genes at days 1 and 2, and 1371 of these genes were still differentially expressed at day 7. The DEGs for each day were clustered with Mfuzz, which identified 8 gene expression clusters with distinct expression profiles across time (Fig. 4B) [24]. Within each cluster, we performed gene enrichment analysis using the DAVID functional annotation tool to identify potential molecular pathways regulated by KDM5 with the kinetic patterns shown in Fig. 4B [29]. The top Uniprot Keyword annotation category for each cluster is presented in Fig. 4C.

As shown in Fig. 4B, clusters 1, 2, and 3 all contain genes that responded to KDM5 inhibition by reducing expression levels during days 0 through 2. Clusters 1 and 3 were enriched with genes that are associated with mitochondrial function (Fig. 4C). Cluster 2 was enriched with genes that are associated with cell cycle function (Fig. 4C), and also included significant enrichment of lipid metabolism genes (*P* = 1.5 E–12, not shown). The remaining gene clusters (4-8) contained genes that increased expression during differentiation in response to KDM5 inhibition (Fig. 4B). These were enriched for genes associated with glycoproteins, metal-binding proteins, and hemostatic proteins (Fig. 4C).

### Mitotic Clonal Expansion in 3T3-L1 Preadipocytes Is Impaired With KDM5 Inhibition

**Figure 5.**
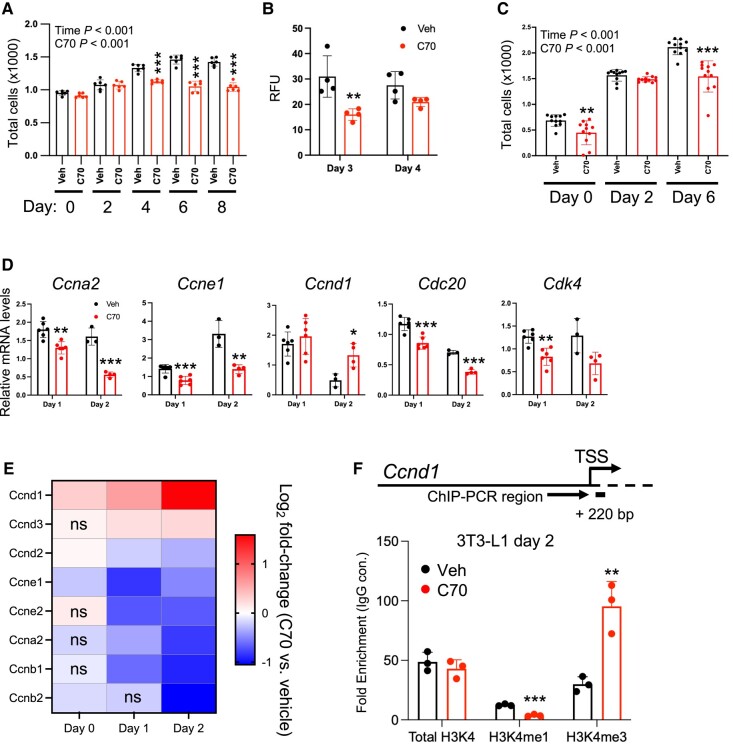
KDM5 inhibition in preadipocytes impairs mitotic clonal expansion. (A) Cell proliferation was assessed in 3T3-L1 preadipocytes treated with C70 or vehicle (Veh) at days −2 to 0, and cells were harvested for analyses on the days indicated. Cell numbers were determined with a hemocytometer after releasing cells with trypsin. (B) Genomic DNA content was measured by fluorescence with Hoechst 33342 dye in 3T3-L1 cells treated as in (A). (C) Brown adipocyte proliferation was assessed by fluorescence with Hoechst 33342 dye after addition of C70 or Veh at days −2 to 0. (D) qPCR analysis of cell cycle–related gene expression in 3T3-L1 cells treated with C70 or Veh at days −2 to 0. (E) Heatmap of differentially expressed cyclin genes during 3T3-L1 differentiation depicting fold-change in expression due to C70 treatment compared to vehicle. Data are from the RNA-seq experiment shown in Fig. 4. ns, not significant. (F) ChIP-qPCR of H3K4 histone methylation status near *Ccnd1* transcription start site (+220 bp) in 3T3-L1 cells. Experiment performed as described in Fig. 3. Data in panels A and C were analyzed by repeated-measures ANOVA, with *P* values for effects of time and C70 treatment shown. Subsequent pairwise comparisons, and analyses in other panels C, D, and F were by *t* test. ** *P* < .01; *** *P* < .001.

The RNA-seq data showing effects of KDM5 activity on cell cycle and mitochondrial gene expression (Fig. 4) led us to directly measure the effect of C70 on these cellular processes. A key event during preadipocyte differentiation is concerted proliferation, known as mitotic clonal expansion, which precedes the induction of adipogenic transcription factor gene expression [30, 31]. To investigate whether KDM5 activity is necessary for preadipocyte proliferation, we treated 3T3-L1 cells with C70 at days −2 through 0, and quantitated cell number and genomic DNA content between days 0 and 4. As determined by repeated-measures ANOVA, C70 significantly reduced cell numbers compared to control treatment, and pairwise comparisons showed reduced cell numbers at days 4, 6, and 8 (Fig. 5A). The reduction in cell number was confirmed as decreased in genomic DNA concentration at days 3 and 4 (Fig. 5B). Interestingly, brown adipocytes treated with C70 at day −1 also showed significant reductions in cell number by repeated-measures ANOVA, with significant reductions by pairwise comparison with control treatment at days 0 and 6 (Fig. 5C).

3T3-L1 preadipocyte expansion relies on cyclin proteins, which are induced in response to cell confluence and components of the adipocyte differentiation cocktail [30, 31]. Our RNA-seq study (Fig. 4) indicated that KDM5 inhibition reduces cell cycle gene expression in 3T3-L1 preadipocytes. We verified this by treating 3T3-L1 cells with C70 at days −2 through 0 and quantifying cyclin gene expression at days 1 and 2 by qPCR. KDM5 inhibition reduced expression of cyclin genes *Ccna2* and *Ccne1* and cell cycle–related genes *Cdk4* and *Cdc20,* but increased levels of *Ccnd1* (Fig. 5D). Our RNA-seq data from 3T3-L1 cells showed that C70 also reduced additional cyclin gene expression (*Ccnb1*, *Ccnb2*, *Ccne2*, *Ccnd2*, *Ccnd3*), and confirmed the increase in *Ccnd1* expression (Fig. 5E). The increase in *Ccnd1* expression was associated with an increase in H3K4me3 marks near the TSS of *Ccnd1* as determined by ChIP-PCR, consistent with direct regulation by KDM5 histone demethylase activity (Fig. 5F).

### Cyclin A2 and E1 Complementation Rescues C70 Impairment of 3T3-L1 Preadipocyte Differentiation

**Figure 6.**
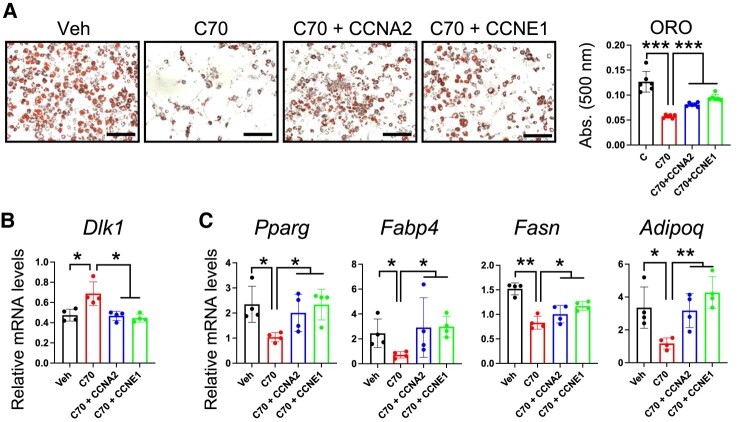
Cyclin A2 and E1 complementation rescues C70 impairment of 3T3-L1 preadipocyte differentiation. 3T3-L1 preadipocytes were transfected at day −3 with expression plasmids for *Ccna2* or *Ccne1* and treated with C70 or Veh on days −2 to 0. (A) Lipid staining with oil red O (ORO) and quantification in 3T3-L1 at day 8 of differentiation. (B) *Dlk1* mRNA levels at day 2. (C) Adipogenic gene expression at day 8. Analysis by ANOVA followed by pairwise *t* test; * *P* < .05; ** *P* < .01; *** *P* < .001.

Our results suggested that cyclins may represent targets of KDM5 action during early stages of preadipocyte differentiation. Given the key role of cyclins in adipogenesis, we tested whether ectopic expression of cyclin A2 (*Ccna2*) or E1 (*Ccne1*) is sufficient to restore adipocyte differentiation in C70-treated cells. 3T3-L1 preadipocytes were transfected with expression vectors for *Ccna2* or *Ccne1* at day −3, treated with C70 or control vehicle at day −2, and collected at days 2 and 8 for analysis. As expected, C70 decreased lipid accumulation at day 8, but cyclin overexpression in C70-treated cells reversed this phenotype (Fig. 6A). Additionally, maintaining either *Ccna2* or *Ccne1* expression normalized C70-induced aberrant expression of *Dlk1* at day 2 (Fig. 6B) and restored expression levels of *Pparg*, *Fabp4*, *Fasn*, and *Adipoq* at day 8 (Fig. 6C). These results suggest that KDM5 regulation of cyclin gene expression is a critical step in adipogenic gene expression and lipid accumulation in differentiating 3T3-L1 adipocytes.

### *Kdm5c* Knockdown Recapitulates Most of the Effects of KDM5 Inhibition on Gene Expression in White and Brown Adipocytes

**Figure 7.**
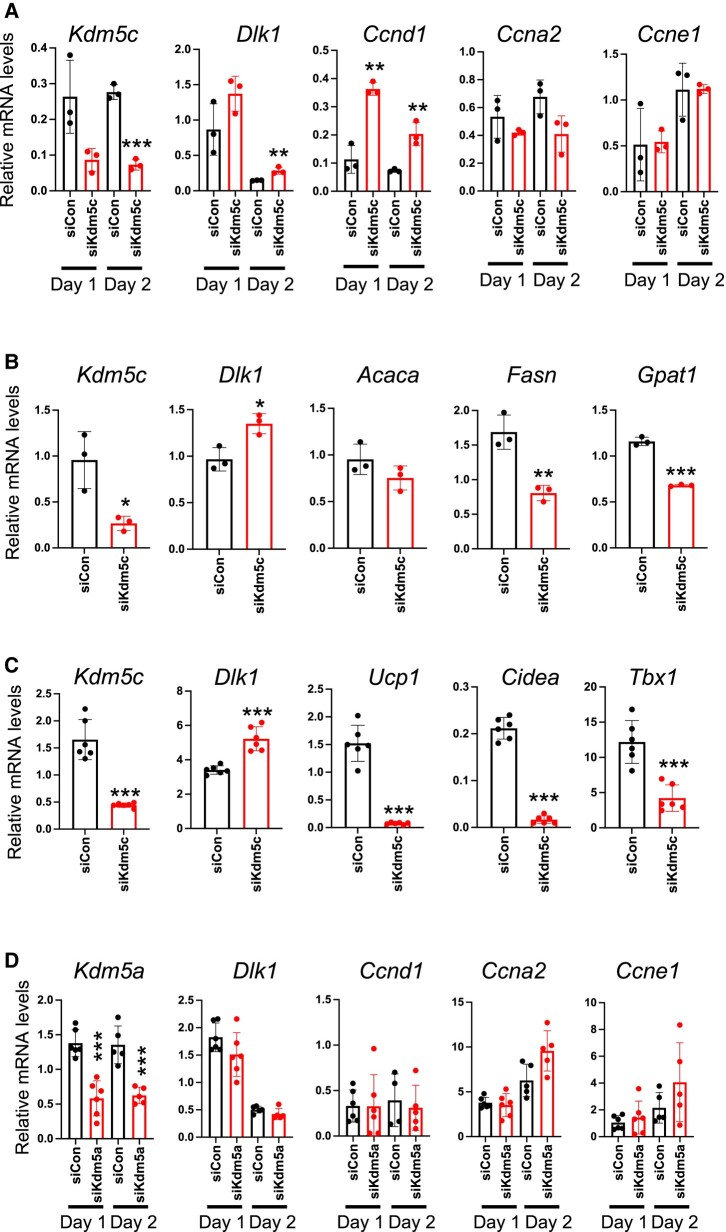
*Kdm5c* knockdown recapitulates effects of C70 on adipocyte differentiation. (A, B) 3T3-L1 preadipocytes were transfected at day −3 with a pool of siRNA directed at *Kdm5c* (siKdm5c) or nonspecific control siRNA (siCon). Gene expression was measured at (A) days 1 and 2 for *Kdm5c*, *Dlk1*, and cell cycle genes, or at (B) day 7 for *Kdm5c*, *Dlk1*, and lipogenic genes. (C) Brown adipocytes were transfected with siKdm5c or siCon at day −1 and *Dlk1* and thermogenic gene expression was assessed at day 7. (D) 3T3-L1 preadipocytes were transfected with siRNA directed against *Kdm5a* or siCon at day −3 and expression determined at days 1 and 2 for *Kdm5a*, *Dlk1*, and cell cycle genes. Data analyzed by ANOVA followed by *t* test; * *P* < .05; ** *P* < .01; *** *P* < .001.

Given that KDM5A and KDM5C proteins (but not KDM5B) are present in preadipocytes (Fig. 1), we hypothesized that the inhibition of one or both of these by C70 might underlie the differentiation-inhibitory effect of C70. To investigate, we performed siRNA knockdown of *Kdm5a* or *Kdm5c* in 3T3-L1 preadipocytes at day −2 and measured gene expression during differentiation. *Kdm5c* knockdown in preadipocytes increased *Dlk1* and *Ccnd1* expression during early differentiation (day 1 and 2) (Fig. 7A), similar to what was observed with C70 treatment (see Figs. 2 and 5). Furthermore, *Kdm5c* knockdown at day −2 prevented attainment of mature adipocyte gene expression at day 7, with aberrant elevation in *Dlk1* levels and failure to induce lipogenic genes such as *Fasn* and *Gpat1* (Fig. 7B). *Kdm5c* knockdown in brown adipocytes also led to aberrant elevations in *Dlk1* gene expression, as well as failure to induce thermogenic genes (*Ucp1*, *Cidea*, *Tbx1*) (Fig. 7C). In contrast, *Kdm5a* knockdown in 3T3-L1 preadipocytes at day −2 did not alter adipogenic gene expression (Fig. 7D). Thus, *Kdm5c* knockdown phenocopies the effects of the C70 KDM5 inhibitor on adipogenic gene expression.

### KDM5 Activity Influences Mitochondrial Function During White and Brown Adipocyte Differentiation

**Figure 8.**
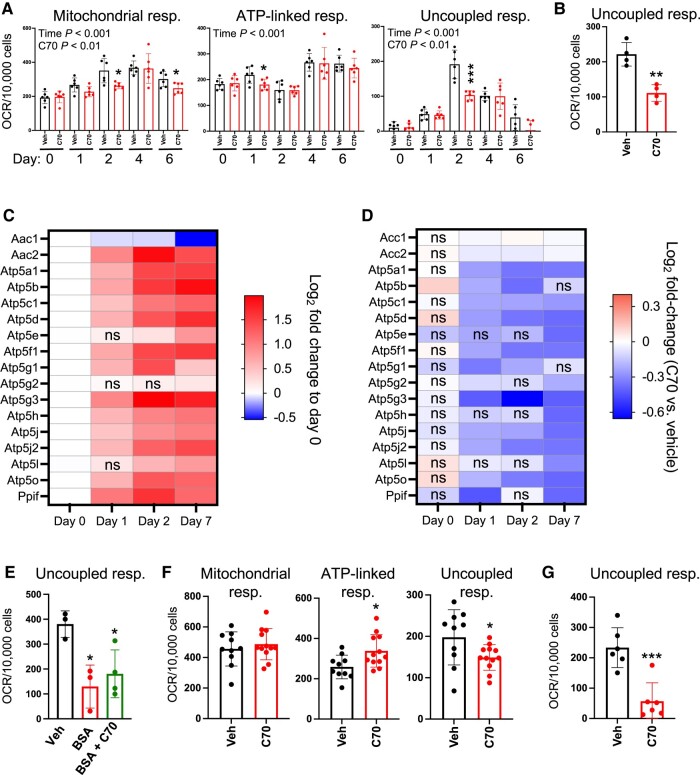
KDM5 inhibition disrupts adipocyte mitochondrial respiration. (A) Total mitochondrial respiration, ATP-linked respiration, and uncoupled respiration were assessed in 3T3-L1 cells at days 0 to 6 of differentiation after C70 or vehicle (Veh) treatment on days −2 to 0. (B) Complex II-driven mitochondrial respiration was assessed using succinate substrate in permeabilized 3T3-L1 cells at day 2. (C) Heatmap of differentially expressed ADP/ATP carrier (AAC) and mitochondrial permeability transition pore (mPTP) complex genes during 3T3-L1 differentiation filtered by fold-change relative to expression at day 0. (D) Heatmap of differentially expressed AAC and mPTP genes during 3T3-L1 differentiation filtered by fold-change in response to C70 treatment. (E) Uncoupled respiration in 3T3-L1 cells at day 2, after Veh, BSA, or BSA + C70 treatment at day 0. (F) Mitochondrial respiration parameters in brown adipocytes at day 5, after C70 treatment at days −1 to 0. Cells were activated with CL316,243 1 hour prior to the assay. (G) Complex II-driven mitochondrial respiration assessed using succinate substrate in permeabilized brown adipocytes at day 5. * *P* < .05; ** *P* < .01; *** *P* < .001.

In the RNA-seq experiment in 3T3-L1 cells, we noted that genes with altered expression levels in response to C70 included pathways associated with mitochondrial function (see Fig. 4C). To investigate whether KDM5 inhibition impacts mitochondrial activity, we treated 3T3-L1 cells with C70 at day −2 and performed bioenergetic studies at day 0 and throughout differentiation. Total mitochondrial respiration increased during 3T3-L1 differentiation and reflected increases in both ATP-linked and uncoupled respiration, as determined by repeated-measures ANOVA (Fig. 8A-8C). C70 treatment led to alterations in total mitochondrial respiration, and this was most evident in uncoupled respiration, as determined by repeated-measures ANOVA, followed by pairwise comparisons between control and C70-treated cells (Fig. 8A and 8C). This indicates that that KDM5 activity is important for the enhanced respiration, and particularly uncoupled respiration, that normally occurs during adipocyte differentiation.

We confirmed that C70 leads to reduced uncoupled respiration in cells that are provided with succinate, a substrate that is used directly by complex II of the electron transport chain (Fig. 8B).

A number of mitochondrial proteins have been associated with free fatty acid-induced mitochondrial uncoupling, including UCP1, ADP/ATP carrier (AAC), aspartate/glutamate carrier (AGC), and the mitochondrial permeability transition pore complex (mPTP) [32-34]. Of these mechanisms for uncoupled respiration, the mPTP and ACC components are expressed in white adipocytes, but *Ucp1* expression occurs primarily in brown adipocytes and AGC genes are expressed in muscle, liver, and heart [35]. Based on tissue distribution, we focused on AAC and mPTP complexes as potential targets of C70 action in white adipocytes.

We retrieved the expression levels from our RNA-seq data from differentiating adipocytes for components of the AAC (*Aac1* and *Aac2*) as well as 15 components of the mPTP complex. *Aac2*, but not *Aac1* mRNA, was induced throughout 3T3-L1 differentiation (Fig. 8C). A strong induction was also observed for the majority of mPTP components at days 1, 2, and 7 compared to day 0 (Fig. 8C). Inhibition of KDM5 with C70 significantly dampened mPTP-related gene expression but had only a slight effect on *Acc1* or *Acc2* gene expression (Fig. 8D).

AAC and mPTP activation is dependent on the presence of free fatty acids [32, 36]. To determine if the uncoupled respiration that was induced during adipocyte differentiation (Fig. 8A) requires fatty acids, we treated 3T3-L1 cells with 0.5% BSA at day 0 to complex free fatty acids and performed respiration studies at day 2. Uncoupled respiration in BSA-treated cells was significantly diminished, indicating that it is enhanced by fatty acids; it was not reduced further by treatment with C70 (Fig. 8E). This suggests that the C70-associated attenuation of uncoupled respiration acts through the same fatty acid-dependent uncoupling complex.

Next, we performed bioenergetic studies with brown adipocytes. Since KDM5 inhibition substantially reduced *Ucp1* expression in differentiated brown adipocytes (Fig. 2H), we expected that uncoupled mitochondrial respiration would also be reduced. To test this, we treated brown adipocytes with C70 at day −1, induced differentiation from day 0 to day 2, and performed bioenergetic measurements at day 5 after treating cells with a beta-adrenergic agonist (CL316,243) for 1 hour. C70 did not alter overall mitochondrial respiration, and slightly enhanced ATP-linked respiration, but as observed in white adipocytes, uncoupled respiration was diminished (Fig. 8F). This is consistent with reduced *Ucp1* expression in response to C70 treatment (Fig. 2H). The decrease in proton leak was confirmed in permeabilized brown adipocytes using succinate as substrate (Fig. 8G).

Thus, the attenuation of mitochondrial respiration gene expression that we observed with C70 treatment of 3T3-L1 cells was reflected in reduced levels of total and uncoupled mitochondrial respiration during 3T3-L1 differentiation. Brown adipocytes also showed reduced uncoupled respiration when KDM5 activity was inhibited. These alterations in mitochondrial function in differentiating adipocytes are consistent with a role for KDM5 in modulating mitochondrial gene expression and function in both white and brown adipocytes.

## Discussion

The regulation of white and brown adipocyte function is crucial for metabolic homeostasis. Regulation of white and brown adipogenic gene expression programs have been widely investigated, but questions remain in several areas, including the epigenetic mechanisms that influence adipose tissue mass and function [37]. Here we demonstrate that KDM5-mediated events are essential for cell proliferation and cellular energetic processes during both white and brown adipocyte differentiation. Previous studies have implicated KDM5 histone demethylases in 3T3-L1 adipocyte differentiation in vitro and in sex-biased adipose tissue expansion in vivo [13, 20]. However, the specific metabolic pathways and cellular functions that are regulated by KDM5 demethylase activity during adipogenesis have not been determined.

By modulating KDM5 histone demethylase activity in a temporally controlled manner using the C70 chemical inhibitor, we determined that KDM5 activity is required in early stages of white and brown preadipocyte differentiation for the establishment of gene expression programs, but it is expendable in mature adipocytes for maintaining cellular lipid content. Consistent with the known role of KDM5 enzymes as H3K4 histone demethylases, we identified key target genes that are regulated at the histone and gene expression level by KDM5 activity. In white preadipocytes, KDM5 activity is critical for the repression of *Dlk1*, which normally occurs as preadipocytes transition to mature adipocytes [38, 39]. Repression of *Dlk1* expression in preadipocytes is necessary for the appropriate induction of cyclin gene expression, which promotes cell proliferation in white preadipocytes prior to differentiation [40]. Expression of *Ccna2* and *Ccne1* were dysregulated when KDM5 activity was inhibited in 3T3-L1 cells. Restoration of cyclin A2 or E1 expression by ectopic expression was sufficient to normalize expression levels of several adipogenic genes that were altered by C70 treatment. Importantly, the cyclin complementation reversed dysregulation of *Dlk1* that was imposed by C70 treatment, allowing cells to progress from preadipocytes to mature adipocytes.

KDM5 activity was also found to have a key role in brown preadipocyte differentiation. As in white adipocytes, KDM5 activity was necessary for *Dlk1* repression and for modulation of H3K4 methylation at the *Dlk1* promoter. Most notably, KDM5 activity was necessary for the establishment of a key property of brown adipocytes—the induction of thermogenic gene expression, including *Ucp1*. H3K4me3 marks at a well-characterized *Ucp1* enhancer located 2.5 kb upstream of the gene were altered in parallel with the gene expression by KDM5 inhibition. Consistent with reduced *Ucp1* gene expression, brown adipocytes treated with KDM5 inhibitor had reduced uncoupled mitochondrial respiration. The interplay between KDM5 inhibition and mitochondrial activity was also evident in white adipocytes. Our unbiased transcriptome analysis in 3T3-L1 adipocytes with KDM5 inhibition revealed dysregulation of genes associated with mitochondrial function. Respiration assays demonstrated that uncoupled mitochondrial respiration normally increases during differentiation of white adipocytes, but this is blunted by KDM5 inhibition. Thus, KDM5 activity regulates uncoupled mitochondrial respiration during both white and brown adipogenesis.

The KDM5 competitive inhibitor employed in our studies binds to the 3 members of the protein family that we detected in adipocytes—KDM5A, KDM5B, and KDM5C [41]. We demonstrated that only KDM5A and KDM5C proteins are present in preadipocytes, which respond to C70, and tested which of these may be critical for the observed effects on adipogenesis by genetic knockdown. *Kdm5c* knockdown in preadipocytes phenocopied the effects of C70 on gene expression, with dysregulation of *Dlk1*, cyclin, and lipogenic gene expression in 3T3-L1 white adipocytes, and dysregulation of *Dlk1* and thermogenic gene expression in brown adipocytes. Conversely, *Kdm5a* knockdown did not alter adipogenic gene expression. We conclude that most of the effects we identified through inhibition of KDM5 activity in preadipocytes are associated with KDM5C function.

KDM5C has been implicated in promoting cancer cell proliferation in multiple cancer types, including breast, prostate, gastric, and colon cancer [42-45]. This has fostered an interest in developing additional inhibitors of H3K4 demethylase activity [41, 46-49]. Our findings indicate that unwanted effects of such inhibitors may include impaired adipogenesis and adipose tissue function. Considering that cancer is often associated with debilitating fat loss [50], it may be important to consider the effects of KDM5 inhibitors on adipose tissue.

In conclusion, we have shown that KDM5A and KDM5C proteins are present in preadipocytes, but not mature adipocytes, and that inhibition of KDM5 histone demethylase activity during a short window of preadipocyte expansion leads to a profound inhibition of adipocyte maturation, lipid accumulation, and mitochondrial respiration program. The KDM5-mediated effect on adipogenesis appears to be driven by the direct regulation of *Dlk1* expression and subsequent regulation of cyclin gene expression. Restoring expression of either cyclin A2 or cyclin E1 is sufficient to overcome the KDM5 inhibition affecting adipogenesis. These findings provide important mechanistic insight into our previous findings that *Kdm5c* gene dosage impacts adiposity in vivo [13].

## Data Availability

The RNA-seq data are deposited in Gene Expression Omnibus (GSE239950).

## Abbreviations

AAC: ADP/ATP carrier
AGC: aspartate/glutamate carrier
ChIP: chromatin immunoprecipitation
DEG: differentially expressed gene
H3K4: histone 3/lysine 4; me, methyl me2, dimethyl; me3, trimethyl; mPTP, mitochondrial permeability transition pore complex
OCR: oxygen consumption rate
qPCR: quantitative polymerase chain reaction
TSS: transcription start site
